# Analysis of Space Debris Orbit Prediction Using Angle and Laser Ranging Data from Two Tracking Sites under Limited Observation Environment

**DOI:** 10.3390/s20071950

**Published:** 2020-03-31

**Authors:** Simon Kim, Hyung-Chul Lim, James. C. Bennett, Michael Lachut, Jung Hyun Jo, Jin Choi, Mansoo Choi, Eunseo Park, Sung-Yeol Yu, Ki-Pyoung Sung

**Affiliations:** 1 Korea Astronomy and Space Science Institute, Daejeon 34055, Korea; hclim@kasi.re.kr (H.-C.L.); jhjo39@kasi.re.kr (J.H.J.); rutcome@kasi.re.kr (J.C.); cmsoo@kasi.re.kr (M.C.); skel93@kasi.re.kr (E.P.); syyu@kasi.re.kr (S.-Y.Y.); kpsung@kasi.re.kr (K.-P.S.); 2 Astronomy and Space Science Department, University of Science and Technology, Korea, Daejeon 34113, Korea; 3 Electro Optic Systems Pty Limited, Queanbeyan, New South Wales 2620, Australia; jbennett@eosspacesystems.com (J.C.B.); mlachut@eosspacesystems.com (M.L.)

**Keywords:** orbit prediction, space debris, electro-optical tracking sensor, laser tracking sensor, ballistic coefficient

## Abstract

The global electro-optical (EO) and laser tracking sensor network was considered to investigate improvements to orbit prediction (OP) accuracy of space debris by combining angle and laser ranging data. However, it is worth noting that weather, schedule and visibility constraints can frequently limit the operations of such sensors, which may not result in sufficient tracking data for accurate OP. In this study, several 1-day OP results for low Earth orbit (LEO) space debris targets were demonstrated under a limited observation environment to verify the OP accuracy through the combination of angle and laser ranging data from two sites. For orbit determination (OD) processes, it was considered to analyze the OP accuracy by one site providing both 2–day arc angle data and 1-day arc laser ranging data, while the other was limited to 1-day arc angle data. In addition, the initial ballistic coefficient ($B_{C}$) application method was proposed and implemented for the improvement of OD/OP accuracy, which introduces the modified correction factor depending on the drag coefficient. In the cases of combining the angle and laser ranging data, the OP results show the 3D position difference values are below 100 m root mean square (RMS) with small position uncertainty. This value satisfies the target OP accuracy for conjunction assessments and blind laser ranging (about 50–100 m at 1000 km altitude). The initial $B_{C}$ application method also shows better OP accuracy than the method without the correction factor.

## 1. Introduction

**Figure 1:**
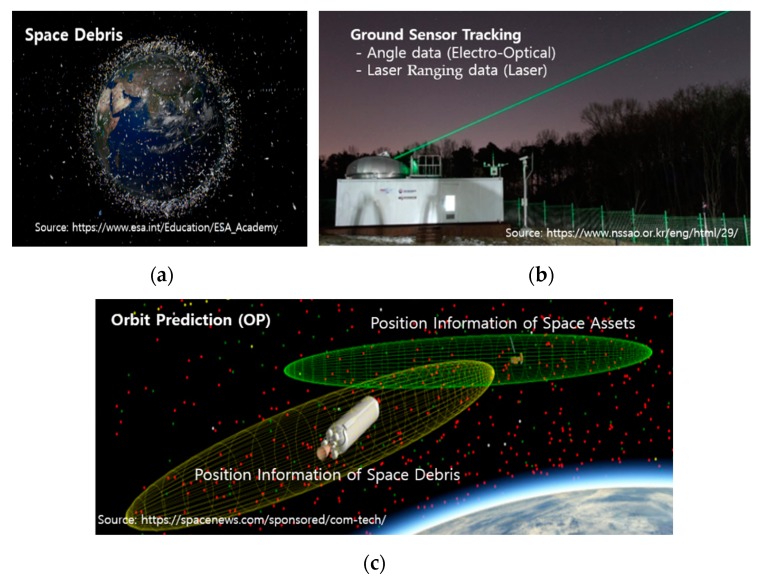
The ground sensor tracking and orbit prediction of space debris. (**a**) space debris; (**b**) ground sensor tracking; (**c**) orbit prediction [2,3,4].

Ground sensor technologies and applications for tracking space debris have received much attention in recent years due to the space environment being congested and clustered by space debris. Valuable space assets such as surveillance, communication and navigation satellite should maintain their orbit position in space to operate properly [1] and avoid the collision from other space objects like space debris. Predicting accurate position information of space debris which is called orbit prediction (OP) is essential not only for the protection of space assets but also for saving their lifetime by reducing unnecessary maneuvers for collision avoidance. Figure 1 shows the visualized concept of ground sensor tracking and orbit prediction of space debris. Various space tracking sensors including radar, electro-optical (EO) and lasers are used to collect tracking data of space debris. Recently, the EO and laser tracking sensors have been widely developed and used for space debris tracking due to their relatively lower cost and high precision compared to radar tracking sensors. Although, it is worth mentioning that operations of such sensors are more limited by weather and visibility conditions, unlike radar tracking sensors, so are complementary in practice.

The EO tracking sensors passively collect the sun-light illuminated by space debris and generate the angle data with about 2–5 arc second accuracy [5]. On the other hand, the laser tracking sensors known as the active sensor transmit laser pulses and then collect the returned signals to calculate the range. Satellite laser ranging (SLR), which is regarded as the representative laser tracking sensor, has many applications including space geodesy and geodynamics. Range measurements are collected by measuring the two-way time of flight to cooperative targets that are equipped with laser retro-reflectors. This technique currently provides sub-cm level ranging accuracy to a subset of orbiting orbits. However, the vast majority of Earth orbiting objects are not equipped with retro-reflectors. Subsequently, some laser tracking sensors began employing high power lasers that can detect returned photon signals from uncooperative targets like space debris.

The global EO and laser tracking sensor network have been considered to improve the OP accuracy of space debris by providing well-distributed and more accurate angle and laser ranging data than that of radar tracking sensors. In theory, the improvement of OP accuracy relies on some factors of the orbit determination (OD) processes related to the condition of tracking data; the amount and type of tracking data, the objects-observer relative geometry, the temporal distribution and accuracy of tracking data [6]. Ideally, the best OP accuracy can be obtained by combining the different types of tracking data which comes from different places if tracking data are dense, well-distributed and of high accuracy like GPS carrier phases [7]. From these perspectives, it is required for multiple EO and laser tracking sensors to be located in different sites which can provide well-distributed combinations of data.

Previous research has shown that huge improvements to OP accuracy was achievable by combining the angle data from the two tracking sites: Swiss Optical Ground Site and SLR site from the international laser range service (ILRS) [6]. However, too much accurate laser ranging data against SLR target (cooperative target) were used for OP accuracy calculation because the laser ranging errors of the uncooperative targets like space debris can be larger than cm level accuracy that is typical of SLR systems, i.e., debris laser ranging systems can yield approximately 1-m level accuracy [Graz: 0.7 m, Shanghai: 0.6–0.8 m, Mt. Stromlo: better than 1.5 m root mean square (RMS)] [8,9,10]. Another research addressed the result of OP accuracy by using real angle and laser tracking data of space debris from one single site to demonstrate the improvement of OP accuracy [11]. But it restricted the achievable result of OP accuracy for the case of one single tracking site and there was also the possibility that sensor bias was not detected, thus further opening investigations on the case of two sites and real space debris laser tracking data for more accurate OP accuracy of space debris is required. To distinguish features of further investigation in this paper from existing researches, the tracking conditions for OP summarized in Table 1. Note that “O” and “X” signified that the tracking conditions were applied or not, respectively.

Practically, the optical sensors are strongly affected by their observation environments which are subject to weather, schedule and visibility constraints. The latter is limited by terminator conditions which can restrict tracking sessions to periods before sun rise and after sun set [12,13]. Since laser space debris tracking sensors usually operate initially with an optical sensor to support acquisition [11], providing OP accuracy of 10–20 arc seconds (about 50–100 m at 1000 km altitude) allows for space debris laser tracking without optical sensors for initial acquisition (blind laser ranging). This allows for the opportunity of operating laser ranging sensors beyond the limitations of terminator conditions which restricts optical tracking sensors operating in the visible light spectrum. This highlights the importance of high accuracy OP of space debris for blinding laser ranging [14]. The application of high accuracy OP for conjunction assessment (CA) is also investigated.

The objective of this study is to verify the OP accuracy improvement corresponding to the above target OP accuracy of 50–100 m at 1000 km altitude by combining the angle and laser ranging data from two tracking sites. The real angle and laser ranging data from multiple EO and laser tracking sensors in Mt. Stromlo and Learmonth sites in Australia are used for the OD/OP process, where the OP results after 1-day are used in two scenarios; (i) to verify CA results and, (ii) initial acquisition for laser ranging to space debris (blind laser ranging). Limited observation environments are considered where one site provides 2–day arc tracking data while the other is limited by 1-day arc tracking data due to restricted conditions.

The OD/OP process is described in Section 2, followed by the ballistic coefficient application to the OD/OP process in Section 3. Finally, the results and discussions are presented in Section 4, followed by conclusions in Section 5.

## 2. Orbit Determination and Orbit Prediction Process

### 2.1. Sites Descriptions

**Figure 2:**
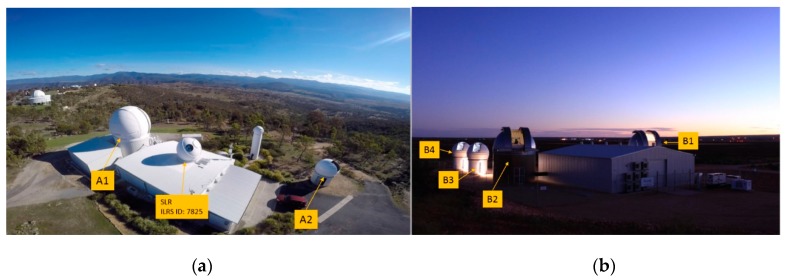
The image of two tracking sites. (**a**) Mt. Stromlo site; (**b**) Learmonth site [15].

The Mt. Stromlo and Learmonth sites in Australia are separately located approximately 3600 km and 35 degrees away in the distance and longitude with very different weather environments. Regarding the data distribution, observations from two sites which have a time difference of approximately 2 h enhances observability conditions for low Earth orbit (LEO) space debris compared to a single site. So, the combined operations of Learmonth and Mt. Stromlo sites expect to give great benefits for tracking space debris [15]. Figure 2 shows the image of two tracking sites in Mt. Stromlo and Learmonth.

### 2.2. Sensor Descriptions

Each site contains multiple EO and laser debris tracking sensors, respectively [Mt. Stromlo: (A1) Active + Passive, (A2) Passive, Learmonth: (B1, B2) Active + Passive, (B3, B4) Passive]. A1, B1 and B2 are optimized for active laser ranging (up to 3000 km altitude depending on size of space debris) but also performed passive optical tracking (up to 70,000 km altitude). And A2, B3 and B4 are optimized only for passive optical tracking (up to 70,000 km altitude) [15,16].

**Figure 3:**
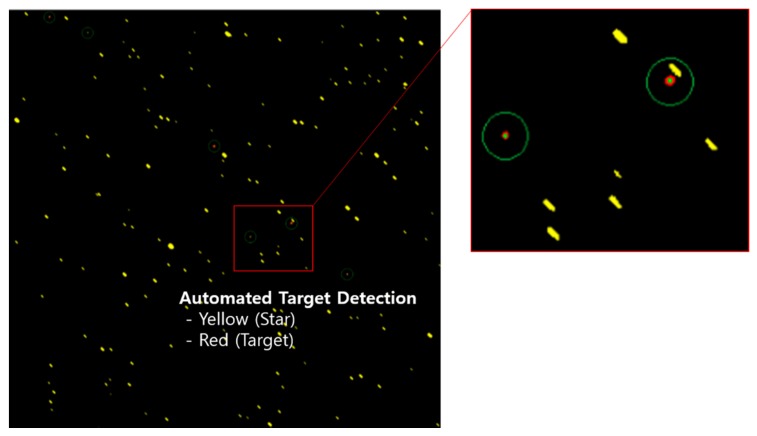
The image of automated target detection in the deep space observation [15].

The core of both sites is the laser space debris tracking sensor. The laser sensor provides laser operating and uses to target acquisition, laser beam lock, delivery, trans and receiver through the telescope. Detailed laser tracking sensor specifications see Table 2. And Table 3 shows configuration of two tracking sites. Passive EO sensor provide excellent pointing accuracy (1–2 arc seconds RMS) and automated target detection capability using astrometric correction comparing known star position. Figure 3 shows the image of automated target detection in the deep space observation. Additionally, the EO and laser sensors can support multiple mission including space debris tracking (against uncooperative targets): Astronomical photometric observations; Astrometric observations; Observation with multiple cameras / fields of view; Satellite laser ranging (against cooperative targets); Using variety of lasers – 1 W class, 100 W class, kW class; 100 Hz – kHz rates; Using variety of detectors – single cell, quad cell, 32 × 32 cell array; Mono-static and bi-static satellite laser ranging; Tracking known targets; Discovery / acquisition of previously-unknown targets via optical detection and streak analysis [15].

### 2.3. Target and Tracking Data Descriptions

Four rocket bodies of space debris were selected to demonstrate the OP accuracy and the proposed initial estimation of ballistic coefficient, which are orbiting lower than 1000 km altitude with a circular orbit. Two rocket bodies of North American Aerospace Defense (NORAD) catalogue ID 4954 and 6276 have spherical shapes, while two targets of NORAD catalogue ID 26703 and 26819 have cylindrical shapes characterized by their length and diameter. The characteristics of selected targets are described in Table 4 and the tracking data achieved by two sites on May 7–9 and May 11–13 in 2019 is shown in Table 5 in terms of data type and tracking date. Note that the selected targets are represented as NORAD catalogue ID number like ID 4954, ID 6276, ID 26703 and ID 26819 in next following Sections.

From Table 5, the EO angle data was obtained from Mt. Stromlo (A1) and Learmonth sites (B2 and B3), while the laser ranging data is employed from only the Learmonth site (B2). Note that the associated tracking site including sensor ID are expressed as A1, B2 and B3 in next following Sections. Note that “⁑” denotes two passes of angle data collected on the associated date from each site. Likewise, the “‡” represents two passes of laser ranging data collected on the associated date.

### 2.4. OD /OP Strategies

Several OD/OP cases are investigated to examine the accuracy and reliability of OP results with the aim to achieve the aforementioned target OP accuracy (about 50–100 m at 1000 km altitude) using the angle and laser ranging data from two sites on the associated date in Table 5. For the preliminary case, 1-day arc angle data from two sites on the 1st date (May 7 for ID 4954, 6276 and 26819, and May 11 for ID 26703) are used to demonstrate the 1 –day OP improvement caused by the geometry dispersion of angle data. Section 4.2 will describe the results of the preliminary case.

Moreover, 3 additional cases are investigated by increasing the number of angle and laser ranging data from the Learmonth site on the 2nd date (May 8 for ID 4954, 6276 and 26819, and May 12 for ID 26703) accompanied with the data on the 1st date (May 7 for ID 4954, 6276 and 26819, and May 11 for ID 26703) used in the preliminary case. To begin, Case 1 will employ 1-day arc angle data from Learmonth on the 2nd date with the data on the 1st date used in the preliminary case (Angle data only). Similarly, Case 2 used 1-day arc laser ranging data on the 2nd date with the data on the 1st date (Angle data + Range data). Case 3 utilize all 1-day arc angle and laser ranging data on the 2nd date with that on the 1st date (Angle data + Range data). The ephemeris of 1-day OP results after the OD span will then be compared with the ephemeris of a reference orbit for assessment (2-day OD + 1-day OP vs 3-day OD). The reference orbit was generated by using all 3-day arc tracking data consisting of angle and laser ranging data from the two sites (3-day OD). More details are described in Table 6.

To determine the initial state, two-line elements (TLE) are used with an assumed initial state uncertainty (Radial: 50 m, in-track: 100 m, cross-track: 20 m). The least square estimator is used to update the initial orbit state and the covariance. Subsequently, a forward sequential filtering and backward smoothing processes are employed [17]. During the entire process, the weight factor of angle and laser ranging data are adjusted according to each sensor’s bias model which provides the distribution of residual ratio within ± 3 sigma. The consistency test between results of the filtering and smothering processes was repeated until its distribution is also within ± 3 sigma. The position difference, 3D position difference RMS, and 3D position uncertainty RMS are used for the OP assessment.

## 3. Ballistic Coefficient Application to the OD/OP Process

The following perturbation models will be employed in the OD and OP process; the gravity effect modeled using the EGM 2008 (70 × 70) gravity model; the solid tide model corresponding to the international Earth rotation service (IERS) conventions was used [18]; the gravitational accelerations by Sun and Moon are only considered for the third body perturbations; and the Jacchia-Bowman 2008 atmospheric density will be used in the drag model. When computing the solar radiation force, the area-to-mass ratio of the space debris will first be determined as $B_{C}$/$C_{D}$ where $B_{C}$ is the initial value of the ballistic coefficient and $C_{D}$ begin the drag coefficient, respectively [19]. Fixing the solar radiation pressure reflectivity coefficient $C_{R}$, 1.0 (solar radiation is absorbed) [20], and taking its product with the pre-determined area-to-mass simply gives the solar radiation force exerted on the space debris of interest for this study.

### 3.1. Initial $B_{C}$ Approximation and $C_{D}$ Determination

In OD and OP processes for space debris targets, the ballistic coefficient ($B_{C}$) measures the susceptibility to drag is a critical parameter to affect the OD accuracy for objects below 1000 km altitude, In some cases, it can be difficult to estimate accurately due to unknown characteristics of the space debris of interest. The $B_{C}$ is defined as

(1) $$B_{C}\;=m/(C_{D}A)$$

where A is the cross-sectional area and m is the mass of the object.

The $B_{C}$ value can be estimated during the OD process. This requires an initial $B_{C}$ value if the individual terms of Equation (1) are unknown or changeable over time [21]. The 2-day arc tracking data from two sites given in this paper are considered to provide a sufficient amount of tracking data for the OD process. This paper proposes the new initial ballistic coefficient ($B_{C}$) application method which introduces the modified correction factor depending on the drag coefficient. The description of the initial $B_{C}$ approximation and $C_{D}$ determination for a proposed initial $B_{C}$ value are presented in the next following paragraphs.

In order to approximate the initial $B_{C}$ value, we can use

(2) $$B_{c}^{*}=12.741621B^{*}$$

where $B_{c}^{*}$ is the drag-like free parameter obtained from a TLE [20].

Sang et al. [22] advised that Equation (2) requires some correction factor since the value of the drag coefficient is only assumed as the nominal $C_{D}$ value of 2.2 in deriving Equation (2). As such, it is recommended to multiply the right-hand-side of Equation (2) with correction factor of 10 or larger. The nominal value of 2.2 which gives the correction factor of 10 is suitable only for the idealistic spherical shape of space objects, but its value is no longer valid for space debris of other shapes like cylindrical, such as that in the case of ID 26703 and ID 26819. In this study, the modified correction factor is calculated from the $C_{D}$ value when the shape and size of the space debris of interest is available. Here we will use a default correction factor of 10 for the nominal value of $C_{D}$ = 2.2. So if there is insufficient information about the space debris object, then this default correction factor is applied. In contrast, for objects of known (cylindrical) shape and dimensions a higher correction factor is used to yield a higher $C_{D}$ value. For instance, if the $C_{D}$ value is assumed to be 2.5, the correction factor will be 11.4 by the ratio (2.5 × 10) ⁄ 2.2 instead of 10.

Pilinski et al. [23] shows the linear relationship between the $C_{D}$ value and the ratio of length and diameter. The linear relationship provides the $C_{D}$ value for the modified correction factor, which shows $C_{D}$ values range from 2.2 to 3.3 corresponding to the ratio of length and diameter from 1 to 3.5. Information of length and diameter for various rocket bodies is provided by Anselmo et al. [24].

### 3.2. Initial $B_{C}$ Value Application

Results of initial $B_{C}$ values considering the modified correction factor in Equation (2) are defined as the corrected $B_{C}$. The original $B_{c}^{*}$ values without the correction factor are defined as calculated $B_{c}^{*}$. Both values are listed in Table 7 with values for the correction factors. For the $B_{c}^{*}$ calculation, the $B^{*}$ value is selected from the TLE data closest to the OD start epoch time to represent the current value of $B_{C}$. A comparison of the OP results when applying the corrected $B_{C}$ value to the case when using the calculated $B_{c}^{*}$ is addressed in Section 4.4.

## 4. Results and Discussions

### 4.1. Residual Ratio and the Filter-Smoother (FS) Consistency Test

**Figure 4:**
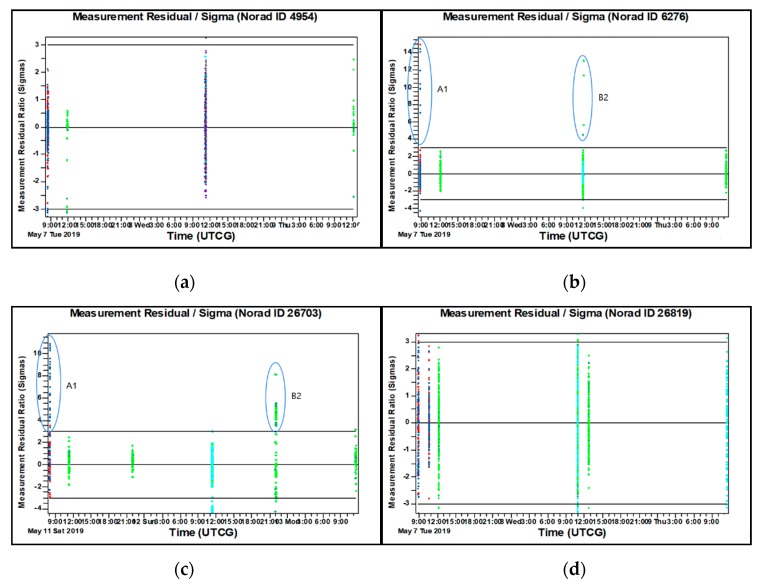
The residual ratio of tracking data, the blue and red from A1 are all angle data, the green and cyan from B2 are angle and laser ranging data, respectively. (**a**) ID4954; (**b**) ID 6276; (**c**) ID 26703; (**d**) ID 26819.

**Figure 5:**
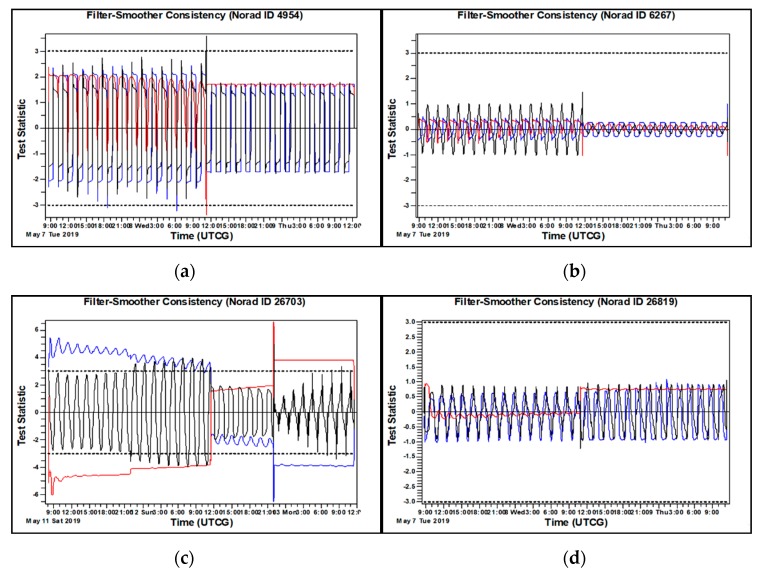
The FS consistency of tracking data, the blue, red and black line represent the radial, in-track and cross-track, respectively. (**a**) ID 4954; (**b**) ID 6276; (**c**) ID 26703; (**d**) ID 26819.

In this section, the results for the OD and OP case studies described above in Section 3 are presented along with discussions. To begin, the reference orbit obtained by using all 3-day arc tracking data is provided to analyze the residual ratio and FS consistency. Figure 4 shows that the results of residual ratio are well-distributed within ±3 sigma after the filtering process. For instance, the results of ID 4954 and ID 26819 show that all tracking data from each site are well-distributed within ±3 sigma in the period of OD processing. However, there are some outliers present in the results for objects with ID 6276 (May 7, May 8) and ID 26703 (May 11, May 13) tracked by A1 and B2. Figure 5 shows that the FS consistency results are also distributed within ±3 sigma excluding the case with object ID 26703, which has small outliers beyond ±3 sigma. These outliers are caused by the tracking data noise corresponding to those outliers present in Figure 4c for the OD residual ratio results. Importantly, notice how but value for the test statistic is temporally attenuated by the laser ranging data in the middle of OD process.

### 4.2. Preliminary Case: the 1-day Arc Angle Data from Two Sites

In this section, the preliminary case was investigated to verify the 1-day OP accuracy improvement by the effect of geometry dispersion from two sites. Section 4.4 will analyze the evaluation of proposed initial $B_{C}$ application. The result of 3D position difference RMS for the 1-day OP assessment is addressed using 1-day arc angle data from two sites and the results are summarized in Table 8. The OD process was performed using 1-day arc angle data of May 7 for ID 4954, 6276 and 26819, and May 11 for ID 26703, and then 1-day OP results were assessed to compare with the OD results obtained by using 2-day arc tracking data. All results show qualitatively that the 1-day OP accuracy can potentially be better than the results using 1-day arc angle data from only one single site (Swiss Optical Ground Site); mean position error 8034 m using 2 passes of angle tracking data [6], but the results do not meet the target OP accuracy set forth in this paper, i.e., a 3D position difference 50–100 m RMS. For objects with ID 6276 and 26703, relatively less accurate OP results are perhaps due to the limited amount of available tracking data (264 and 110, respectively) while some of that data are excluded in the OD process because of the tracking data noises from A1 on May 7 and May 11.

### 4.3. Results of OP Cases

**Figure 6:**
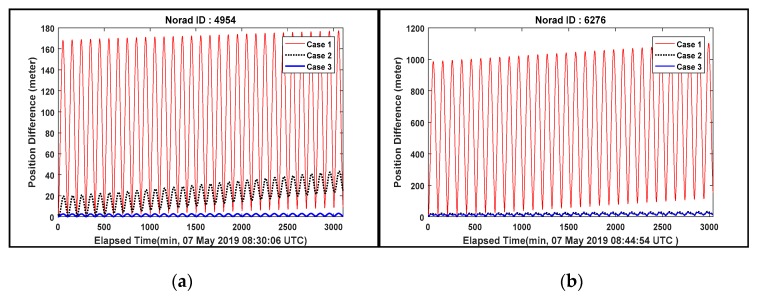

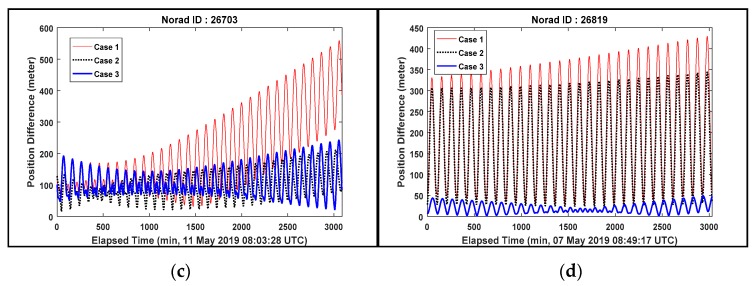
Position difference results of Case 1, 2 and 3. (**a**) ID 4954; (**b**) ID 6276; (**c**) ID 26703; (**d**) ID 26819.

Figure 6 shows the position difference results for the three cases to analyze the variation of OP accuracy assessment compared to the reference ephemeris generated by the 3-day arc OD process (2-day OD + 1-day OP vs 3-day OD). In contrast to Case 1, the results of Case 2 and 3 show the consistent or small increments with small position difference values over the entire ephemeris period.

**Figure 7:**
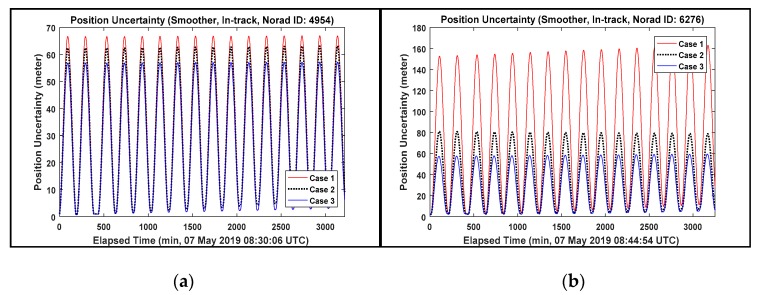

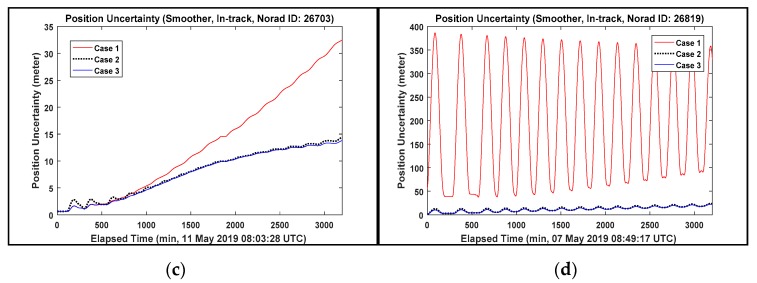
The position uncertainty of Case 1, 2 and 3. (**a**) ID 4954; (**b**) ID 6276; (**c**) ID 26703; (**d**) ID 26819.

Table 9 summarizes the results of 3D position difference RMS as well as 3D position uncertainty RMS. And the smothering results of position uncertainty corresponding to the in-track direction which provides dominant errors among the radial, in-track and cross-track (RIC) direction are presented in Figure 7 to analyze the variation of position uncertainty value.

It is summarized from Table 9 and Figure 7 as follow:

- Most results of OP accuracy for Case 2 and 3 show that the 3D values of position difference are below 100 m RMS when the angle and laser ranging data are combined. In summary, taking into consideration near circular orbits and apogee altitude of space debris approximately below 1000 km described in Table 4, 2-day arc tracking data combining the angle and laser ranging data can achieve the target OP accuracy.
- The triangulation effect and accurate range by combining the angle and laser ranging data can provide more statistical confidence in all directions of orbit state. All results of OP accuracy for Case 2 and 3 in Table 9 show that the 3D values of position difference are below 50 m RMS. Most position uncertainty variation for the elapsed time in Figure 7 also displayed stable and consistent fluctuations, however, the ID 26703 shows a slight increased tendency of position uncertainty in Case 2 and 3 since the results of the FS consistency includes some outliers deviating from ±3 sigma provided by the tracking data noises from A1 and B2. (see Figure 4 in Section 4.1).
- The maximum 3D values of position difference RMS are 614.43 m (ID 6276), 180.22 m (ID 26819) and 118 m (ID 26703) for Case 1, 2 and 3, respectively. The tracking data noises from A1 and B2 affect the results of ID 6276 and 26703. But the result of ID 26819 in Case 2 is caused by the 2 passes of angle data (766) from B2 (May 8–9) which exist next to the laser ranging data among all tracking data. If the OD results of the reference orbit are dominated by these angle data, this position difference value (180.22 m) is achievable because the 1-day OP result of Case 2 would be dominated by the laser ranging data. This is further supported by the results of Case 3 (ID 26819) which includes these angles data (766) next to the laser ranging data. The results show a significantly decreased position difference value (21.96 m RMS).
- The minimum 3D values of position difference RMS are 101.67 m (ID 4954), 17.9 m (ID 6276) and 1.34 m (ID 4954) for Case 1, 2 and 3, respectively. The accurate results from Case 1 and 3 (ID 4954) were caused not only by using the tracking data collected from the 3 tracking sensors in two sites (A1, B2, and B3) but also from the tracking data well-distributed within ±3 sigma from A1 and B3 during the OD processing period (May 7–9). The accurate result of Case 2 (ID 6276) is caused by the 2 passes of laser ranging data. The OD results of the reference orbit and 1-day OP result of Case 2 are also dominated by the accurate laser ranging data. The results of Case 3 for object ID 6276 further supports this reasoning. The Case 3 result (4.83 m RMS) shows similar results of Case 2 (17.9 m RMS).

### 4.4. Ballistic Coefficient Evaluation

**Figure 8:**
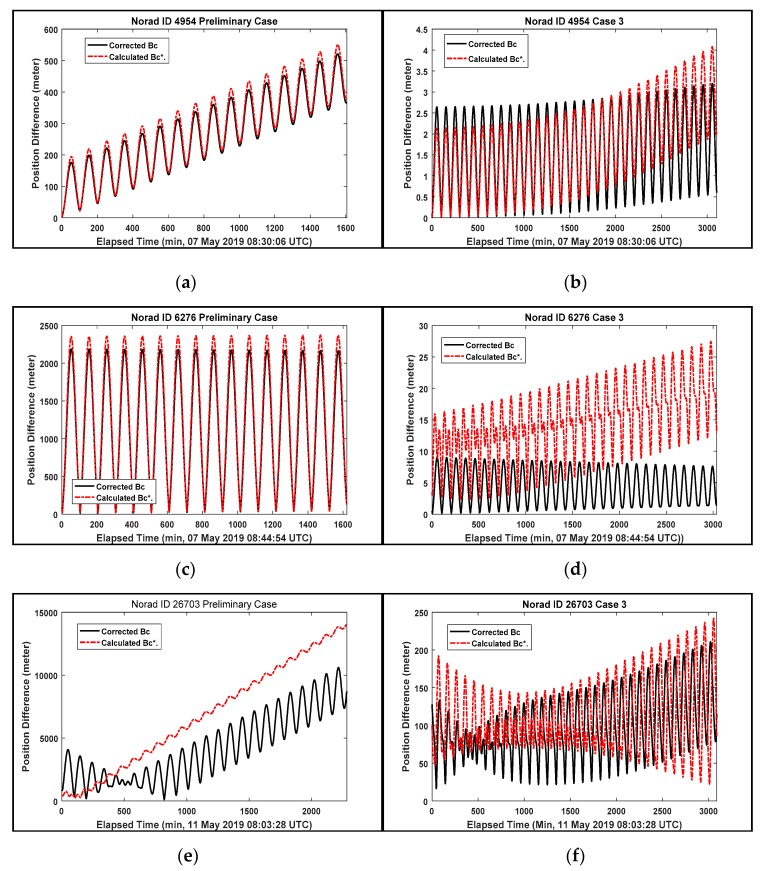

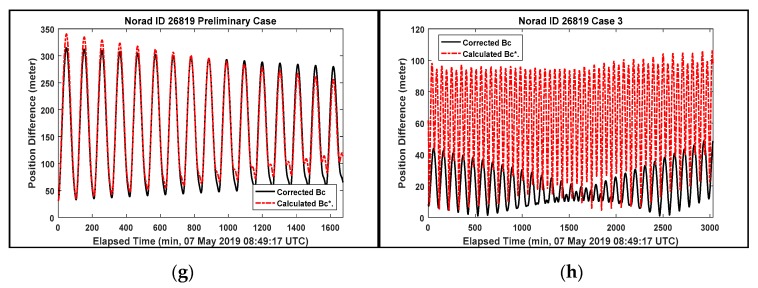
The position difference of the corrected $B_{C}$ and calculated $B_{c}^{*}$ application. (**a**) ID 4954, preliminary Case; (**b**) ID 4954, Case 3; (**c**) ID 6276, preliminary Case; (**d**) ID 6276, Case 3; (**e**) ID 26703, preliminary Case; (**f**) ID 26703, Case 3; (**g**) ID 26819, preliminary Case; (**h**) ID 26819, Case 3.

Sang et al. [14] evaluated the initial $B_{C}$ through the comparison of OP accuracy results by applying a new method to calculate the initial ballistic coefficient. In this section, the results of OP accuracy are presented to evaluate the proposed method to compute the initial $B_{C}$ value by introducing the modified correction factor depending on the drag coefficient. To investigate the difference of OP accuracy results depending on the amount and arc length of tracking data, the OP accuracy results of two cases obtained by the 1-day arc tracking data and the 2-day arc tracking data from two sites are assessed. The preliminary case and Case 3 were selected as the 1-day arc tracking data and 2-day arc tracking data case, respectively to analyze the OP accuracy depending on the amount and arc length of tracking data. Note that the tracking data of Case 3 consists of both angle and laser ranging data. Figure 8 and Table 10 shows the variation of position difference and 3D position difference result for the preliminary case and Case 3 corresponding to the NORAD IDs.

It is summarized from Figure 8 and Table 10 as follows:

- The position difference results in Figure 8 demonstrate that the corrected $B_{C}$ value as the initial ballistic coefficient provides better OP results than the calculated $B_{c}^{*}$ value. The results of OP accuracy improvement in Table 10 show that the corrected $B_{C}$ application provides maximum improvement values of 2964.69 m (ID 26703) in preliminary case and 50.99 m (ID 26819) in Case 3. These improvements are caused by applying the modified correction factor values (11.4 and 12.7, respectively) from high drag coefficient value corresponding to the cylindrical shape of ID 26703 and ID 26819 given in the proposed method. So the corrected $B_{C}$ value computed by the proposed method provides a more accurate way to represent the atmospheric drag and the shape of space debris than the calculated $B_{c}^{*}$.
- Case 3 with 2-day arc tracking data shows a significantly smaller position difference value than the preliminary case with 1-day arc tracking data in Figure 8. However, for the object with ID 26819 in the preliminary case, Figure 8g shows the results of position difference values according to the initial $B_{C}$ value application are reversed toward the end epoch time. Although the modified correction factor values are considered from the high drag coefficient value corresponding to the cylindrical shape of ID 26819, the tumbling effects generated by the high ratio of length and diameter may bring frequent variation of $B_{C}$ (the $B_{C}$ sensitivity of a rocket body) [25].
- To achieve better OP accuracy for requiring high OP accuracy such as the CA and blind laser ranging, the corrected $B_{C}$ value provides more stable and accurate OP results given sufficient tracking data.

## 5. Conclusions

This study analyzed the achievable 1-day OP results for space debris to verify the effect of combining the angle and laser ranging data from two sites. The multiple EO and laser sensors located at the various sites have different observation environment depending on the weather, visibility and schedule constraints. The limited observation environment was considered to provide 2-day arc angle data and 1-day arc laser ranging data from the Learmonth site and only 1-day arc angle data from the Mt. Stromlo site. The OP accuracy results of several cases were investigated by using the different combination of angle and laser ranging data. The cases using the angle and laser ranging data together (Case 2, 3) satisfied the target OP accuracy (about 100 m at 1000 km altitude) required for CA and blind laser ranging. In addition, a new method was proposed to compute the initial $B_{C}$ value, which introduces the modified correction factor depending on the drag coefficient obtained from the ratio of length and diameter. It was also demonstrated that this proposed initial $B_{C}$ application provided better OP accuracy than the method without the correction factor. The specific drawback of the proposed method for the initial $B_{C}$ application comes from the inaccuracy of TLE information since its computation always starts from the $B^{*}$ value. And unlike this limited environment scenario, the best environment scenario using the angle and laser ranging data from two or multiple stations is desired to investigate. This will show the maximum OP accuracy results for the another mission area like debris laser maneuvering which can change the orbit of space debris to avoid collisions.

## Figures and Tables

**Table 1:** The tracking conditions for OP.

Tracking Conditions	Existing Research [6]	Existing Research [11]	Further Investigation
Combining Angle and Laser Ranging Data	O	O	O
Tracking from Two Sites	O	X (Single Tracking Site)	O
Targeting to Real Space Debris Target	X (SLR Target)	O	O

**Table 2:** The laser tracking sensor specification [15].

Parameters	Specification	Laser Sensor Module
Type	Solid-state Nd YAG	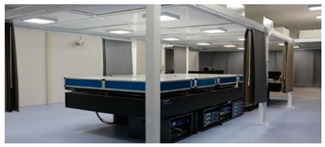
Average Power	1 kW	
Repetition Rate	200 Hz	
Pulse Width	15 ns	
Pulse Energy	Up to 5 J	
Range Accuracy	~ 1.5 m RMS	
Pointing Accuracy	2 arc seconds RMS	

**Table 3:** The configurations of two tracking sites [15].

Sensor ID	Tracking Site	Aperture	Configuration
A1	Mt. Stromlo	1.8 m	Active + Passive
A2	Mt. Stromlo	0.7 m	Passive
B1	Learmonth	1.0 m	Active + Passive
B2	Learmonth	1.0 m	Active + Passive
B3	Learmonth	0.7 m	Passive
B4	Learmonth	0.7 m	Passive

**Table 4:** The description of space debris targets in LEO.

	NORAD ID	Description	Eccentricity	Perigee (km)	Apogee (km)	Mass (kg)	Length (m)	Diameter (m)	RCS (m^2^)
1	4954	THOR BURNER 2 R/B	0.0037618	744.6	798.4	116	0.8	0.7	1.7004
2	6276	THOR BURNER 2 R/B	0.0037044	801.7	855	116	0.8	0.7	1.1577
3	26703	SL-18 R/B	0.0005144	581.2	588.3	300	2.5	1.4	2.4311
4	26819	SL-8 R/B	0.0024131	969	1004.5	1435	6	2.4	6.7097

**Table 5:** The number of tracking data for the OD process (May 7–9, May 11–13, 2019).

NORAD ID	Tracking Site	Sensor ID	May 7		May 8		May 9		May 11		May 12		May 13	
			Angle	Angle		Range	Angle	Range		Angle	Angle	Range		Angle
4954	Mt. Stromlo	A1	330											
	Learmonth	B2		620 ⁑		68								
		B3	40				38							
6276	Mt. Stromlo	A1	264											
	Learmonth	B2	264	494		56	222							
26703	Mt. Stromlo	A1								110				
	Learmonth	B2								118	274 ⁑	187 ‡		58
26819	Mt. Stromlo	A1	370 ⁑											
	Learmonth	B2	262	766 ⁑		116		90						

**Table 6:** The descriptions of OD/OP Cases with the OP accuracy assessment.

Cases	Tracking Site	Tracking Data Type			
		1-Day arc	2-Day arc	3-Day arc	OP Accuracy Assessment
Reference Orbit (Angle + Range)	Mt. Stromlo	Angle			OD result ephemeris including subsequent tracking data (3-day arc)
	Learmonth	Angle	Angle or Laser ranging	Angle or Laser ranging	
Preliminary Case (Angle only)	Mt. Stromlo	Angle	1 day OP result ephemeris comparing Case 3 OD result ephemeris		
	Learmonth	Angle			
Case 1 (Angle only)	Mt. Stromlo	Angle		1 day OP result ephemeris comparing reference orbit ephemeris	
	Learmonth	Angle	Angle		
Case 2 (Angle + Range)	Mt. Stromlo	Angle			
	Learmonth	Angle	Laser ranging		
Case 3 (Angle + Range)	Mt. Stromlo	Angle			
	Learmonth	Angle	Angle and Laser ranging		

**Table 7:** The list of initial $B_{C}$ application.

NORAD ID	Mass (kg)	Length (m)	Diameter (m)	L/D Ratio	$C_{D}$	Calculated $B_{c}^{*}$	Correction Factor	$\mathrm{Corrected}\;B_{C}$
4954	116	0.8	0.7	1.14	2.26	0.000231	10.23	0.00235
6276	116	0.8	0.7	1.14	2.26	0.000193	10.23	0.00198
26703	300	2.5	1.4	1.79	2.5	0.000384	11.4	0.00438
26819	1435	6	2.4	2.5	2.8	0.000518	12.7	0.00658

**Table 8:** The 3D position difference (m) RMS of 1-day arc angle data from two sites.

NORAD ID	4954	6276	26703	26819
3D	290.24	1199.26	5242.23	157.83
Radial	27.06	342.08	589.34	49.77
In-Track	288.91	1140.43	5172	145.23
Cross-Track	6.42	143.62	619.73	36.64

**Table 9:** The 3D position difference RMS and 3D position uncertainty RMS of Case 1, 2 and 3.

NORAD ID	Case	Position Difference / RMS (m)				Position Uncertainty / RMS (m)			
		3D	Radial	In-Track	Cross-Track	3D	Radial	In-Track	Cross-Track
4954	1	101.67	27.48	97.77	4.65	35.47	9.79	33.87	3.91
	2	21.94	3.29	21.67	0.77	39.99	11.06	37.94	6.14
	3	1.34	0.34	1.29	0.07	29.93	8.30	28.55	3.43
6276	1	613.43	160.62	589.89	50.19	95.25	25.19	89.38	21.2
	2	17.9	3.47	15	9.13	44.67	13.14	42.53	3.7
	3	4.83	1.51	4.59	0.04	32.4	9.28	30.83	3.65
26703	1	240.31	50.54	230.81	43.83	32.07	0.99	32.03	1.17
	2	103.63	25.52	89.14	46.29	15.41	0.96	15.34	1.14
	3	115.62	45.43	101.55	31.49	10.49	1.03	10.29	1.80
26819	1	213.56	59.83	202.93	29.16	209.22	63.61	198.4	19.07
	2	180.22	51.01	170.5	28.38	14.81	2.67	14.36	2.44
	3	21.96	6.23	20.78	3.42	14.04	2.89	13.49	2.6

**Table 10:** The 3D position difference RMS of preliminary case and Case 3.

NORAD ID	Case	L/D Ratio $\left(C_{D}\right)$	Correction Factor	3D Position Difference/RMS (m)		
				$①\;\mathrm{Corrected}\;B_{C}$	$②\;\mathrm{Calculated}\;B_{c}^{*}$ (W/O Correction Factor)	OP Accuracy Improvement (②–①)
4954	Preliminary Case	1.14 (2.26)	10.23	290.24	300.86	10.62
	Case 3			1.34	1.71	0.37
6276	Preliminary Case	1.14 (2.26)	10.23	1199.26	1303.24	103.98
	Case 3			4.83	14.63	9.8
26703	Preliminary Case	1.79 (2.5)	11.4	5242.23	8206.92	2964.69
	Case 3			115.62	128	12.38
26819	Preliminary Case	2.5 (2.8)	12.7	157.83	164.88	7.05
	Case 3			21.96	72.95	50.99

## Abbreviations

$B_{C}$	Ballistic Coefficient
CA	Conjunction Assessment
$C_{D}$	Drag Coefficient
EO	Electro-Optical
FS	Filter-Smoother
IERS	International Earth Rotation Service
ILRS	International Laser Range Service
LEO	Low Earth Orbit
LRA	Laser Retro-reflector Array
NORAD	North American Aerospace Defense
OD	Orbit Determination
OP	Orbit Prediction
RMS	Root Mean Square
SLR	Satellite Laser Ranging
TLE	Two-Line Element
